# Parents of young infants report poor mental health and more insensitive parenting during the first Covid-19 lockdown

**DOI:** 10.1186/s12884-022-04618-x

**Published:** 2022-04-09

**Authors:** Marion I. van den Heuvel, Stefania V. Vacaru, Myrthe G. B. M. Boekhorst, Mariëlle Cloin, Hedwig van Bakel, Madelon M. E. Riem, Carolina de Weerth, Roseriet Beijers

**Affiliations:** 1 Department of Cognitive Neuropsychology, Tilburg University, Tilburg, The Netherlands; 2 Donders Institute for Brain, Cognition and Behavior, Radboud University Medical Center, Nijmegen, The Netherlands; 3 Department of Medical and Clinical Psychology, Tilburg University, Tilburg, The Netherlands; 4 Tranzo, Tilburg University, Tilburg, The Netherlands; 5 Behavioral Science Institute, Radboud University, Nijmegen, The Netherlands; 6 Clinical Child & Family Studies, Faculty of Behavioral and Movement Sciences, Vrije Universiteit, Amsterdam, The Netherlands

**Keywords:** COVID-19, Parenting, Abuse, Fathers, Mothers, Stress

## Abstract

**Background:**

The Covid-19 pandemic has put an unprecedented pressure on families with children. How parents were affected by the first Covid-19 lockdown during the early postpartum period, an already challenging period for many, is unknown.

**Aim:**

To investigate the associations between Covid-19 related stress, mental health, and insensitive parenting practices in mothers and fathers with young infants during the first Dutch Covid-19 lockdown.

**Methods:**

The Dutch Covid-19 and Perinatal Experiences (COPE-NL) study included 681 parents of infants between 0 and 6 months (572 mothers and 109 fathers). Parents filled out online questionnaires about Covid-19 related stress, mental health (i.e. anxiety and depressive symptoms), and insensitive parenting. Hierarchical regression models were used to analyze the data.

**Results:**

Parents of a young infant reported high rates of Covid-19 related stress, with higher reported stress in mothers compared to fathers. Additionally, the percentages of mothers and fathers experiencing clinically meaningful mental health symptoms during the pandemic were relatively high (mothers: 39.7% anxiety, 14.5% depression; fathers: 37.6% anxiety, 6.4% depression). More Covid-19 related stress was associated with more mental health symptoms in parents and increased insensitive parenting practices in mothers.

**Conclusions:**

The results emphasize the strain of the pandemic on young fathers’ and mothers’ mental health and its potential negative consequences for parenting. As poor parental mental health and insensitive parenting practices carry risk for worse child outcomes across the lifespan, the mental health burden of the Covid-19 pandemic might not only have affected the parents, but also the next generation.

## Background

The Covid-19 pandemic has put an unprecedented pressure on people worldwide [1, 2], including families with children. Many parents faced drastic changes in their daily family routine, such as a shift to working at home, closure of daycare, unavailability of grandparents, and teaching older siblings at home [3]. Accumulating evidence indicates the subsequent strain on the mental health of parents all over the world. In Europe, for example, increased parenting stress and exhaustion was found in Spanish parents [4] and Italian parents [5, 6]. Reports from Asia also show significantly increased parenting stress during the lockdown period in Japan [7], Singapore [8] and Israel [9]. A recent report from a large longitudinal cohort in Canada showed increased depression and anxiety in mothers as compared to three earlier pre-pandemic time points [10]. How parents are affected during the early postpartum period, which in itself is an already challenging period for many, is still largely unknown. Here, we investigated the associations between Covid-19 related stress, mental health, and insensitive parenting in mothers and fathers with young infants.

The birth of a child often brings much joy and happiness, but also challenges parents’ available resources. The many biological, social and psychological changes during the transition to parenthood and the postpartum period can trigger parental mental health problems [11]. For example, hormonal changes [12], sleep issues [13], psychosocial stress [14], and the adaption to parenthood [15] are known for their potential to trigger symptoms of anxiety and depression in parents during the postpartum period (for a detailed review, see [16, 17]). The first months postpartum are also a critical period for key developmental processes for the infant [18–20]. As such, young infants are susceptible to environmental influences, including the quality of care they receive. One of the most central elements of high-quality care is a parent’s ability to be sensitive [21, 22], which refers to the extent to which a caregiver is available and aware of the infant’s needs, and able to respond to them timely and adequately [23]. A large body of research has shown a strong and consistent association between parental mental health issues and insensitive parenting practices, such as emotional unavailability and harsh parenting (e.g., physical punishment; for a review, see [24]). Importantly, these associations were not only found at clinical levels of psychopathology, but also in community samples [24]. Mental health problems thus not only affect the parents, but likely also the infant through negative changes in parenting [25]. Though several researchers and clinicians have raised concerns about the mental health of postpartum women and their families during the Covid-19 pandemic [26], it is as yet unclear how the Covid-19 outbreak and lockdown affected the mental health and caregiving of parents during the already intensive and challenging postpartum period.

H Prime, M Wade and DT Browne [27] present a conceptual model to explain how the Covid-19 outbreak can impact the child through changes in parental wellbeing and caregiving quality. The model theorizes that the Covid-19 outbreak and restrictions, such as social distancing and confinements, induce stressful alterations in social support and financial security of parents. In turn, these Covid-19 related stressors increase parental mental health symptoms, which will affect family functioning as a whole, including parent-child interaction quality. The current study follows this theoretical model of Prime and colleagues [27] and aims to determine the impact of the Dutch Covid-19 outbreak and lockdown on Covid-19 related stress, mental health, and parenting in mothers and fathers with a young infant. Based on previous research and theoretical work on the Covid-19 impact on families [24, 27], we expected that higher Covid-19 related stress would increase mental health problems and insensitive parenting practices in both mothers and fathers.

## Materials and methods

### Participants

**Fig. 1:**
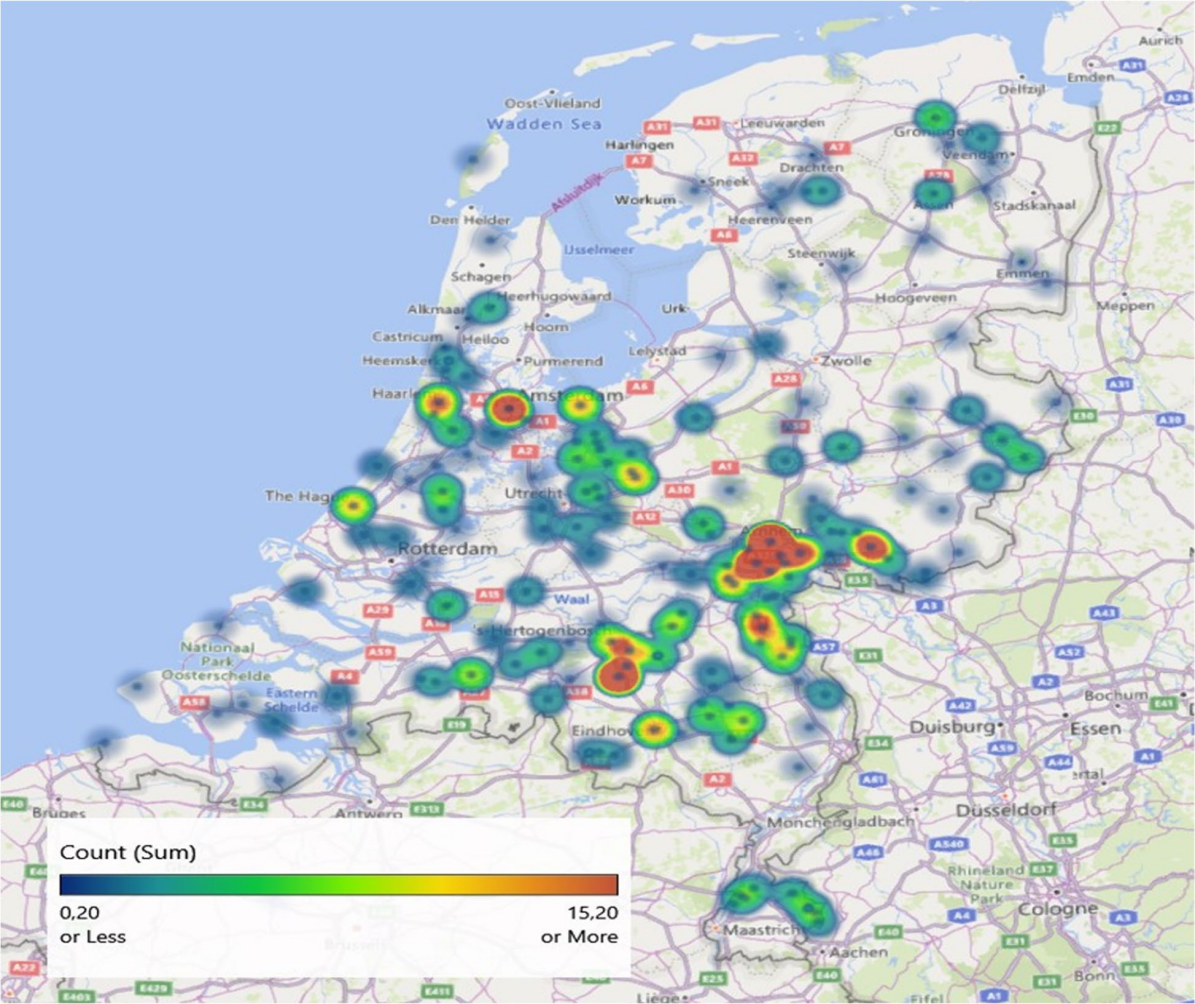
Distribution map of COPE questionnaire respondents. Note that Covid-19 affected the South of the Netherlands first and that the regions with higher numbers of respondents correspond to higher populated areas. This Figure was generated with the 3D map add-on in Excel 2016

This study is part of an ongoing international research alliance investigating perinatal experiences of (expecting) parents during the Covid-19 outbreak and their children: the CovGen Research Alliance (www.covgen.org). More information about the study background and used questionnaires can be found at our OSF page (https://osf.io/uqhcv/). In the Netherlands, both women and men with a current wish to become parents, who were expecting a child or who had a baby of 6 months or younger were recruited as part of our Covid-19 Perinatal Experiences (COPE-NL) study. Participants were recruited via multiple sources, including social media (52.3%), mouth-to-mouth (18.6%), email (e.g., from their midwives; 17.8%) and other (11.4%). Respondents’ geographical distribution reveals a national spread (see Fig. 1 for a heat map of the Netherlands with the participant distribution). The questionnaire data was collected at the peak of the Dutch Covid-19 outbreak and first lockdown (4 April-10 May 2020). The restrictions to prevent the spread of the Coronavirus in the Netherlands at that time included closures of schools and day care centers, remote working, social distancing, closure of restaurants and sport clubs, and cancellation of large events. Dutch people were allowed to leave their home and shops were allowed to stay open but people were advised to keep social distance during this (self-proclaimed) ‘intelligent lockdown’.

For the purpose of this study, the parents who had a 0–6-month-old infant were selected (N_Mothers_ = 683 N_Fathers_ = 129). Parents with missing data on our main variables (i.e., Covid-19 related stress, mental health, and insensitive parenting practices) were excluded (83 mothers and 11 fathers). Finally, data was checked for suspicious responding patterns, including extremely fast completion time (e.g., less than 5 min to complete full questionnaire) and extremely low responding variation (e.g., always responding with “1”). Based on these criteria, 28 additional mothers and 9 additional fathers were excluded. The final sample consisted of 681 parents, including 572 mothers (*M* = 31.4 years, *SD* = 4.1) and 109 fathers (*M* = 32.9 years, *SD* = 4.7). At the time of the investigation, the mean age of the infants was 2.1 months (0–6 months; *SD* = 1.7), with 78% of the infants being full-term (gestational age ≥ 37 weeks). Most parents were living together (97.5%) and about half of the sample (47.3%) had no other children living in the household (besides the baby), see Table 1. Most participants had no immigration background, with about 9.6% of the participants reporting that they or their (grand)parents were not born in the Netherlands. Socioeconomic status (SES) and educational levels varied considerably, with about 1/3 of parents having completed lower or medium education and 21.2% of parents reporting incomes lower than €40.000 annually (below this annual income, parents can apply for social benefits).

**Table 1:** Sample characteristics for mothers and fathers separately

	Mothers				Fathers			
	N	%	Mean (SD)	Range	N	%	Mean (SD)	Range
**Descriptives**								
Country of birth								
The Netherlands	504	90,5			96	89,8		
The Netherlands, but parents not	13	2,3			3	2,8		
The Netherlands, but grandparents not	10	1,8			1	,9		
Other than the Netherlands	30	5,4			7	6,5		
Marital status								
Living with partner	543	94,9			108	100		
Other	17	5,1						
**Covariates**								
Parent age (years)**	545		31,40 (4,14)	19–45	100		32,85 (4,73)	23–50
Infant age (months)	570		2,09 (1,71)	0–6	108		2,00 (1,60)	0–6
Level of education*								
Low	16	2,8			3	2,8		
Medium	152	26,6			36	33,6		
High	391	70,6			68	63,6		
Annual household income								
< €40,000	127	26,6			14	14,6		
€40,000 to €100,000	304	63,6			73	76,1		
> €100,000	47	9,8			9	9,3		
Number of children in the household								
1	258	45,1			59	54,1		
2	227	39,7			35	32,1		
3 or more	74	15,2			13	13,8		
**Study variables**								
Component 1: General Covid-19 worries***	467		5,45 (2,29)	0–10	93		3,99 (2,48)	0–9,65
Component 2: Work and finances worries	505		4,70 (2,99)	0–10	100		4,29 (2,77)	0–9,80
Component 3: Social support worries**	506		3,38 (2,59)	0–10	101		2,60 (2,47)	0–9,50
Postpartum depression (EPDS)***	560		6,65 (4,98)	0–26	108		4,42 (4,28)	0–19
Postpartum anxiety (STAI)	564		38,66 (13,69)	20–146	109		37,07 (13,53)	20–131
Insensitive parenting	569		1,28 (,43)	1–2,73	109		1,24 (,37)	1–2,73

* = p < .05, ** = p < .01, *** p < .001*,* Level of education = low: primary education or secondary pre-vocational education, medium: secondary education or vocational education, high: Bachelor or Master’s degree or higher (i.e. PhD)

At the time of questionnaire completion, 44.1% of the parents knew at least 1 person that had tested positive for Covid-19, and almost 10% (8.2%) of the parents reported that they knew more than 5 confirmed Covid-19 cases. While 22.5% of our participants reported to currently have Covid-19 symptoms (e.g., dry cough, sore throat, fever, shortness of breath), only 2 participants (0.3%) had been tested positive themselves. Note that at that time Covid-19 tests were unavailable to the general public in the Netherlands, resulting in most people with symptoms never being tested. Regarding work, 36% of the parents reported to have experienced a change towards working from home, and almost half of the parents (49.8%) reported that their partner started working from home. While only very few parents lost their job (3.2%), 11.5% of the parents reported lower job security and 15.6% reported higher work demands. Furthermore, about one third of the parents experienced disturbances concerning childcare availability (33.5%).

All sample characteristics are presented in Table 1. All participants independently and anonymously filled in the questionnaires. Therefore, we are unable to merge the data and connect couples. Because it was also not possible to assume that the data from mothers and fathers were completely independent (i.e., not from couples), we analyzed the data for the mothers and fathers separately in the subsequent analyses.

### Procedure

Participants enrolled in the study by clicking on the questionnaire link in the advertisement, email, or website and reading the information letter. After providing online informed consent, the questionnaire started. The questionnaire was programmed in Qualtrics (www.qualtrics.com) and took approximately 30–45 min to complete. After completion, the participant was asked whether they wanted to receive an online €10 gift voucher for their effort. To prevent participants from responding to the questionnaire more than once, we restricted the questionnaire to one response per IP address. If participants tried to fill out the questionnaire again, this response was flagged and participants could not complete the questionnaire. Via the Qualtrics software, we also prevented indexing which means that search engines were blocked from including our questionnaire in their search results. The participants were asked for consent to be approached for future research.

The Ethics Review Board of Tilburg University [RP2019–143] approved the study, which was conducted according to the Declaration of Helsinki. All participants provided online informed consent before completing the survey.

### Questionnaires

#### Stress related to the Covid-19 outbreak

In total, eight questions were asked about stress related to Covid-19 outbreak and aspects of daily life that the Covid-19 outbreak impinged on (see Table 2). Items were answered on a Visual Analogue Scale (VAS) scale (slider) from 0.0 to 10.0. The items were part of a newly developed instrument, the Covid-19 and Perinatal Experiences (COPE) questionnaire, for the purpose of this study by the authors and international collaborators of the CovGen Research Alliance [28].

**Table 2:** Results of the factor analysis for Covid-19 related stress items

	Item	Component 1: General Covid-19 worries	Component 2: Work and financial worries	Component 3: Social support worries
1	In general, what is the level of distress you have experienced due to Covid-19 related symptoms or potential exposures you have had?	0.924		
2	In general, what is the level of distress you have experienced due to Covid-19 related symptoms or potential exposures your family and friends have had?	0.953		
3	In general, what is the level of distress you have experienced due to the employment and financial impacts of the Covid-19 outbreak?		1.002	
4	In general, what is the level of distress you have about future employment and financial impacts of the Covid-19 outbreak?		0.935	
5	In general, what is the level of distress you have experienced with disruptions in the support you receive from your partner due to the Covid-19 outbreak?			0.780
6	In general, what is the level of distress you have experienced with disruptions to your social support due to the Covid-19 outbreak?			0.900
7	In general, what is the level of distress you have experienced in taking care of your family and child due to the Covid-19 outbreak?	0.571		0.329
8	Please indicate your overall level of stress related to the Covid-19 outbreak.	0.539		0.349
	Explained variance (%)	56.23%	12.86%	11.69%

Only correlations above *r* = 0.30 are shown

To identify potential latent factors underlying the Covid-19 related stress items, we first ran a factor analysis (FA) on the full sample, including data from all participants of the COPE study (https://cope-study.com/). Prior to performing the FA, the suitability of the data for factor analysis was assessed. The data met the assumptions for performing FA: inter-item correlations exceeding .30 [29], no correlation coefficient exceeded .90 (i.e. no problem of multicollinearity), the determinant of the correlation matrix (.007) was greater than Fisher’s threshold (.00001), the Kaiser-Meyer-Oklin value (.835) was higher than the recommended value of .60 [29, 30], and Barlett’s Test of Sphericity reached statistical significance (*p* < .001) [31].

Subsequently, inspection of the scree plot and the component matrix indicated three latent components, which together explained 80% of the variance. To check whether the three-component solution also represented the parent subsample of the COPE cohort, the three components were extracted from the parent sub-dataset through the Principal component analysis (PCA) method with Oblimin rotation (see Table 2). The 8 items loaded well on the three factors and the statistics were similar to the full sample. The three factors are: General Covid-19 worries (Component 1), Work and financial worries (Component 2), Social support worries (Component 3). Subsequently, individual scores for the three components were created by averaging the respective items, but only when no more than one item was missing. Higher scores reflect more worries.

#### Parental mental health

##### Edinburgh postnatal depression scale

Depressive symptoms were measured with the Dutch translation [32] of the self-report 10 item Edinburgh Postnatal Depression Scale (EPDS [33];). Items were scored on a 4-point Likert scale, from 0 to 3. Higher scores reflect higher depressive symptoms. Cronbach’s alpha in the current study was α = 0.86 for mothers, and α = 0.86 for fathers). To describe the percentage of mothers at risk for depression, a cut-off EPDS score of 10 was used, as is recommended for screening purposes in the general population (during the postnatal period) by primary care workers [33]. Men are suggested to be less emotionally expressive, compared to women, and thus would score lower on the EPDS in case of the same level of distress as women [34–36]. We therefore followed the procedure as reported by [37] and used a lower cut-off score of 9 for fathers.

##### State-trait anxiety inventory

Postnatal anxiety symptoms were measured with the Dutch translation [38, 39] of the 20-item state anxiety subscale of the State-Trait Anxiety Inventory (STAI [39];). Items were measured on a 4-point scale, with higher scores reflecting more anxiety. Cronbach’s alpha in the current study was α = 0.86 for mothers and α = 0.86 for fathers. A cut-off score of ≥40 was used to describe the percentage of parents at risk for postpartum anxiety [40].

#### Insensitive parenting practices

To measure insensitive parenting practices among our parents we asked about the frequency of three types of insensitive parenting practices. These three items were: *during the past seven days, including today, how often: 1) were you emotionally less or not available for your child(ren), 2) did you have verbal outbursts towards your child(ren)?,* and *3) did you have physical outbursts towards your child(ren)?* These items were constructed by the researchers to quickly assess three key facets of insensitive parenting: emotional unavailability, emotional abuse, and physical abuse. Items were scored from 1 (not at all) to 5 (very often). Emotional unavailability and verbal outbursts were relatively common, with 26.3% of parents reporting they were emotionally unavailable to some extent (scores of > = 2), and 36% of parents reporting some extent of verbal outbursts in the past 7 days (scores of > = 2). However, physical outbursts towards the child(ren) were only reported sporadically (3.4%; scores of > = 2). Because items were intercorrelated (*r*’s ranging between .31 and .58, all *p*-values <.001), scores were subsequently averaged, with one item maximally allowed to be missing. The Cronbach’s alpha (α = 0.68) showed an acceptable internal consistency of the scores. Higher scores on this composite score reflect higher insensitive parenting.

### Statistical approach

First, the variables were checked for outliers. The 14 outliers, defined as >3SD above the mean, on the insensitive parenting variable were winsorized (i.e. replaced by value of +3SD above the mean). When checking the normality of the variables, the insensitive parenting variable was skewed. However, as transformations were not able to solve this skewness and the residuals of the regression models were normally distributed, the original variable was kept. Additionally, VIF values were checked for multicollinearity (all were close to 1). We ran Independent T-tests to compare the levels of Covid-19 related stress, mental health problems, and insensitive parenting between mothers and fathers. Subsequently, we investigated whether parental mental health in our sample was affected by the pandemic by comparing the prevalence of depression and anxiety, using the cut-off scores. We then computed Pearson’s correlations to determine the association between all three components of Covid-19 related stress, mental health, and insensitive parenting.

For the main analyses investigating how Covid-19 related stress predicted mental health and insensitive parenting, hierarchical regression models were computed for mothers and fathers separately. We selected covariates based on studies indicating that SES and number of children living in the household were associated with harsh parenting [41, 42], and that SES and parental age were risk factors for postpartum mental health problems [43]. The following covariates were included in Step 1: age of infant, age of parent, SES (average of z-scores (one missing allowed) of parental education level and annual income of the family; *r*_*mothers*_ = .49 *r*_*fathers*_ = .39; all *p-values* < .001), and number of children living in the household. The Covid-19 related stress components were entered in Step 2.

Power analyses computed with G*Power [44], and based on multiple regression analysis with 7 or less predictors, revealed a power of >.90 to detect small effects (f2 = 0.02) in the sample of mothers (*N* = 572) and medium effects (f2 = 0.15) in the sample of fathers (*N* = 109). This study lacks the power to detect small effects in the sample of fathers (<.50).

## Results

### Covid-19 related stress

**Fig. 2:**
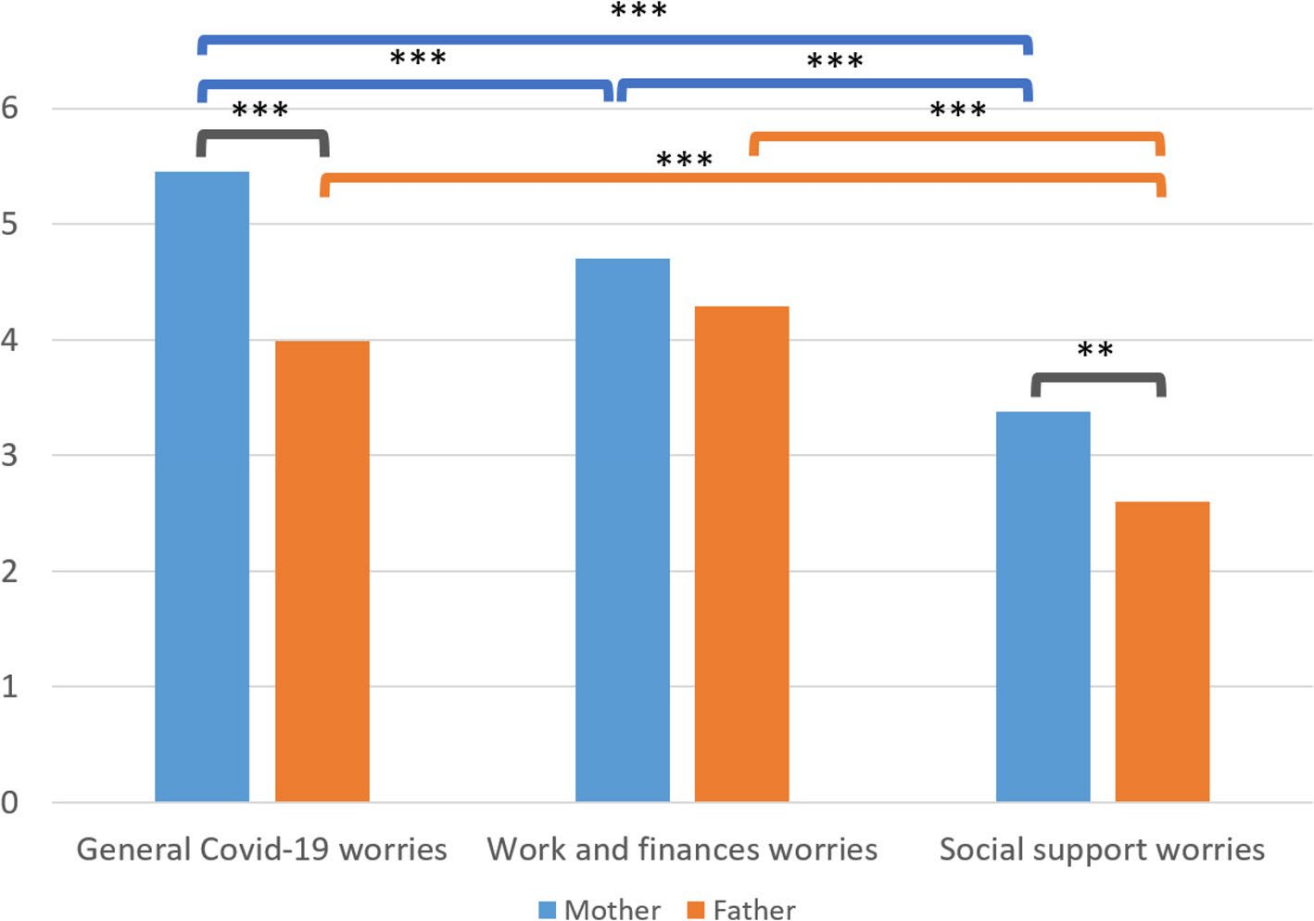
Graphical overview of mean differences on Covid-related worries between mothers and fathers. The black lines indicate significant differences between mothers and fathers, while the blue and orange lines represent within group differences for mother and fathers, respectively. Note. *** = *p* < 0.001; ** = *p* < 0.01

When asked about their main source of stress due to the Covid-19 outbreak, most mothers reported that their main stress source originated from being isolated (23.9%), while most fathers reported that financial worries were their main stress source (15.7%). Furthermore, more fathers reported to experience no stress (16.7%), compared to mothers (6.2%). Independent T-tests revealed that mothers and fathers experience comparable levels of stress related to the impact of Covid-19 on work and finances (*t* = 1.270, *p* > .05), but that mothers experience significantly more stress related to general Covid-19 related worries (*t* = 5.549, *p* < .001), and to social support worries (*t* = 2.779, *p* < .01). One-way repeated measures ANOVA’s with post-hoc comparisons using Tukey HSD tests indicated that mothers experienced significantly more general Covid-19 worries, followed by work and financial worries, followed by social support worries (all *p*’s < .05). Fathers experienced the most general Covid-19 worries and work and financial worries (*p* > .05), followed by social support worries (*p* < .05). Thus, both mothers and fathers experienced social support worries the least compared to the other two types of Covid-19 related worries. See Table 1 for the respective means and standard deviations and Fig. 2 for an overview of the mean comparisons.

### Parental mental wellbeing

For depression, clinical levels of symptoms were reported by 14.5% of the mothers (using an EPDS cut-off score of ≥10), and 6.4% of the fathers (using an EPDS cut-off score of ≥9). For anxiety, clinical symptoms were reported by 39.7% of the mothers, and 37.6% of the fathers (using a STAI cut-off score of ≥40). The clinically relevant anxiety rates were not significantly different for mothers and fathers [*X*^*2*^ *=* 0.264*, p* > .05], but for depression, significantly more mothers reported clinically relevant scores than fathers [*X*^*2*^ *=* 5.402*, p* < .05].

### Insensitive parenting practices

When examining differences on the composite insensitive parenting between mothers and fathers, no significant differences were found (*t* = .917, *p* > .05).

### Covid-19 related stress impact on parental mental health and insensitive parenting practices

The Pearson correlations between the study variables (the three Covid-19 stress components, parental depression and anxiety, and harsh parenting practices) can be found in Table 3, separated for mothers (lower half) and fathers (upper half). The correlations indicate that the three Covid-19 stress components are all inter-correlated in both mothers and fathers (all *p*-values <.001), and that these three components are related to higher depression and anxiety in both mothers and fathers (all *p*-values <.001). In mothers, these three components are also related to more insensitive parenting (all *p*-values <.01). In fathers, general Covid-19 worries and social support worries are related to more insensitive parenting (*p* = .031, and *p* = .006 respectively), but not for work and financial worries related to the Covid-19 pandemic (*p* = .051).

**Table 3:** Correlations between the study variables, separated for fathers and mothers

	1	2	3	4	5	6
1. Component 1: General Covid-19 worries		.70**	.63**	.51**	.49**	.22*
2. Component 2: Work and finance worries	.55**		.44**	.327**	.42**	.20^+^
3. Component 3: Social support worries	.55**	.47**		.449**	.59**	.27**
4. Anxiety symptoms	.49**	.34**	.38**		.54**	.29**
5. Depression symptoms	.50**	.33**	.49**	.62**		.44**
6. Insensitive parenting	.14**	.13**	.24**	.29**	.41**	

lower half = mothers, upper half = fathers

* *p* < .05, ** *p* < .01, ^+^ *p < .10*

The results of our hierarchical linear regression models (see Table 4) indicate that for mothers, more general Covid-19 related worries and social support worries significantly predicted more postnatal symptoms of anxiety and depression, even after controlling for covariates. Additionally, maternal postnatal anxiety was also predicted by work and financial worries. In contrast to mothers, only social support worries were significantly associated with anxiety and depression for fathers, whereas the other two types of Covid-19 related worries did not associate with paternal mental health. The full model including the three factors of Covid-19 related worries explained 36.5% of the anxiety symptoms experienced by mothers and 24.2% of the anxiety symptoms experienced by fathers, and 26.4% of the depression symptoms experienced by mothers and 44.8% of the depression symptoms experienced by fathers.

**Table 4:** Hierarchical linear regression models of Covid-related stress, parental symptoms of anxiety and depression, and insensitive parenting

		Parental Anxiety				Parental Depression				Insensitive Parenting			
		B	β	R^2^ model	R^2^ change	B	β	R^2^ model	R^2^ change	B	β	R^2^ model	R^2^ change
**Mothers**													
Step 1	Age parent	.089	.129			.079	.069			.004	.034		
	Age infant	.084	.285			.026	.010			.011	.043		
	Number of children	1130	.634			.427	.073			.193	.359***		
	SES	−.122	.227	.016		−.173	−.082	.020		.008	.041	.144	
Step 2	General Covid-19 worries	1774	.278***			.669	.318***			−.003	−.017		
	Work and financial worries	.559	.202**			.098	.063			.013	.091		
	Social support worries	.839	.242**	.365	.394***	.400	.205**	.264	.245***	.031	.174**	.191	.047***
**Fathers**													
Step 1	Age parent	.148	.459			.106	.146			.007	.081		
	Age infant	−.341	1189			.246	.111			.031	.123		
	Number of children	−.928	2789			.550	.126			.177	.358*		
	SES	−.375	1104	.028		−.369	−.189	.198		.000	.001	.233	
Step 2	General Covid-19 worries	2473	1091			.120	.092			−.006	−.038		
	Work and financial worries	−.312	.884			.209	.178			.012	.092		
	Social support worries	.939	.961*	.242	.214**	.508	.341**	.448	.250***	.019	.112	.253	.020

* *p* < .05, ** *p* < .01, ^***^ *p < .001*

For insensitive parenting, worries about social support predicted more insensitive parenting in mothers only. As our parenting variable did not discriminate between insensitive parenting towards the infant and/or other children within the family, we ran sensitivity analyses for the group of first-time mothers (47%) and multiparous mothers (53%), separately. In both groups, more social support worries predicted significantly more insensitive parenting in mothers (first-time mothers: *r* = .230, *p* = .001, 4.7% variance explained; multiparous mothers: *r* = .278, *p* < .001, 10.3% variance explained). However, when we ran sensitivity analyses in the full hierarchical regression model (controlling for the effects of types of covid worries on each other), only the model for multiparous women yielded a significant effect: maternal social support worries predicted more insensitive parenting. In the model for first-time mothers, 4.7% of the variance in insensitive parenting was explained. In the model for the multiparous mothers, 10.3% of the variance in insensitive parenting was explained. For fathers, the three components of Covid-19 related stress were not significantly associated with insensitive parenting after controlling for covariates.

## Discussion

The aim of the current study was to examine the associations between Covid-19 related stress, mental health, and insensitive parenting practices in mothers and fathers with a young infant during the first Dutch lockdown. First, we observed that anxiety and depression symptoms in young parents were high, especially for anxiety. Almost 40% of the mothers and 40% of the fathers reported anxiety symptoms above the clinical cut-off. Next, we identified three types of Covid-19 related stress in parents: general worries about Covid-19, worries about work and finances, and worries about disruptions in social support. We observed differences between mothers and fathers in the types of Covid-19 related stress and worries they experienced. While mothers mostly experienced general Covid-19 worries, fathers experienced mostly work and financial worries. Both mothers and fathers experienced social support worries the least compared to the other two types of worries. Next, we found that more social support worries were associated with more symptoms of anxiety and depression in mothers and fathers. For mothers only, more general Covid-19 worries were related to more symptoms of anxiety and depression, and more work and financial worries were related to more symptoms of anxiety. Our correlational analyses indicated that general Covid worries and social support worries were associated with more insensitive parenting practices. However, after controlling for confounding variables, only higher social support worries were associated with more insensitive parenting in mothers, but not in fathers.

The percentages of mothers and fathers that meet the criteria for clinical levels of depression and anxiety in our pandemic sample are high. Compared to recent work in a large Dutch perinatal sample *before* the pandemic, which used similar cut-offs [45], the percentage of parents who reported clinical anxiety is indeed clearly higher during the lockdown (37.6–39.7% versus 30–31% pre-pandemic). Likewise, compared to other work in Dutch postnatal samples *before* the pandemic [46], we observed higher rates of clinically relevant depressive symptoms in mothers during the lockdown (14.5% versus 5.9–8.7% pre-pandemic). For fathers, less pre-pandemic data on depressive symptoms is available. However, the recent study from M Missler, A van Straten, J Denissen, T Donker and R Beijers [47] reported that 13.9% of the fathers scored above the cut-off score of ≥9 for postpartum depressive symptoms, which is higher than the percentage in our COPE sample (6.4%). The observation that parents with young infants experienced more symptoms of anxiety and depression during the pandemic, as compared to pre-pandemic samples, is in line with previous studies reporting worse mental health during the pandemic in pregnant women (e.g., [48–50]) and parents of older children [51]. Our report adds to these findings by showing that symptoms of anxiety and depression are also higher in mothers and fathers during the first few months postpartum. The transition to parenthood and the early postpartum period are already associated with increased mental health issues [52–54]. Our results indicate that the Covid-19 pandemic may add additional pressure to this challenging period, leading to even more mental health problems in mothers and fathers.

Furthermore, marked differences were found in the types of Covid-19 stress experienced by mothers and fathers. Firstly, mothers experienced more general Covid-19 worries and social support worries compared to fathers, indicating that mothers worry more about their family (including themselves) getting infected with the virus and about disruptions in social support due to the pandemic. Additionally, when asked about their main source of stress, mothers reported social distancing as the main stressor, while fathers reported worries about work and finances as the main stressor. These findings could be the result of the traditional role of the father as the family’s main financial provider. In the Netherlands, mothers often switch to a part-time job after having children and become the main caregiver, while fathers continue working full-time [55]. Remarkably, while worries related to social support were relatively the lowest in both mothers and fathers, these worries comprised the strongest predictor for worse parental mental health and maternal insensitive parenting practices. This finding is in line with research showing that a healthy social support network is essential for new parents to deal with the struggles of early parenthood [56, 57], and emphasize the need for attention and care for isolated families with a young infant during the pandemic.

We found that increased worries about social support during the pandemic were associated with more symptoms of anxiety and depression in mothers and fathers. Other studies also reported associations between Covid-19 related worries and anxiety and/or depression in parents of older children (e.g., [5, 7, 51, 58, 59]). However, according to a recent ecological momentary assessment study, parents of adolescents seemed to deal fairly well with the changes related to the Covid-19 pandemic [60]. In this last study, parents’ negative affect was not impacted by Covid-19 stressors, such as contact with Covid-19 impacted individuals, having relatives with Covid-19, or helping their adolescent with schoolwork. It is thus possible that family mental health and functioning is mostly compromised by the pandemic and associated lockdown in families with infants and (younger) children, potentially because these children are more dependent on their parents for care and regulation. Also, the biological, social and psychological changes and challenges that belong to the transition to parenthood and postpartum period may make parents of infants and young children more susceptible to the negative impact of the pandemic.

For mothers only, other types of Covid-19 related stress were also related to increased mental health problems during the lockdown. More general Covid-19 worries were related to more symptoms of anxiety and depression, and more work and financial worries were related to more symptoms of anxiety. This result is in line with a recent brief report, showing that mothers with an infant in the NICU during the pandemic experienced more mental health issues when they scored higher on Covid-19 related health worries [61]. It thus appears that social support worries seemed to affect the mental health of both mothers and fathers during the first Dutch lockdown, while general Covid-19 related worries and worries about work and finances also impacted the mental health of mothers.

Interestingly, after controlling for confounding variables, higher social support worries were related to more insensitive parenting in mothers only. The sensitivity analyses indicated that these results hold for primiparous women as well as for multiparous women but appear stronger in multiparous women. The social support worries were characterized by the level of distress experienced by disruptions in the support received from the partner and from the social network. Possibly, mothers perceived lower levels of support in childcare and parenting from their partners and social network, which in turn, could become a burden and increase insensitive parenting. As in the Netherlands parental tasks and caregiving duties are more often the responsibility of mothers compared to fathers [55], this could explain why social support worries were associated with more insensitive parenting in mothers, but not fathers. Nonetheless, it is also possible that we missed a genuine effect in fathers due to lower power. Our sample of fathers is considerably smaller than our sample of mothers, leading to lower power to detect small effects in fathers (see Statistical Approach section). However, our sample of fathers was large enough to detect medium effects, and the effect sizes for the fathers were much lower compared to mothers, indicating a genuine non-significant effect for fathers.

Although not the focus of the current paper, we observed that family size matters for insensitive parenting during the lockdown. The more children in the household, the higher the frequency of insensitive parenting practices in both mothers and fathers. This finding is in line with research into risk factors of child maltreatment [42], which also identified the number of children in the household as a risk factor. As such, these findings indicate that special consideration should be given to large families and those families with multiple children at home during lockdown measures, to protect infants and their siblings against potential insensitive parenting and parental abuse.

### Strengths and limitations

The data of this study were collected real time through online questionnaires at the peak of the first Dutch Covid-19 outbreak and lockdown, covering the whole country. Also, we included both mothers and fathers in our study. However, there are also limitations to note. First, because of the cross-sectional design, it is not possible to draw any conclusions about causal effects. For example, we do not know whether maternal mental health problems lead to insensitive parenting or vice versa. Longitudinal data from our cohort are currently being collected; these data will give us the opportunity to investigate trajectories over time to help unravel potential causal effects. Secondly, more mothers than fathers enrolled in the study, as is common in parenting studies including fathers [62]. As a result of the discrepancy between the sample sizes of mothers and fathers, our power to find effects for the group of fathers was lower compared to the group of mothers. Relatedly to the previous point, all mothers and fathers independently and anonymously filled in the questionnaires. Therefore, we were unable to merge the data and connect couples. Thirdly, we were unable to distinguish between insensitive parenting towards the infant versus other (possible) children in the household. Though sensitivity analyses in the group of first-time mothers versus multiparous mothers indicated similar results, future research should investigate the parenting quality towards each child separately. Finally, our completely anonymous approach for recruiting participants, mainly via social media, made it impossible to gather any information about eligible mothers and fathers who declined participation. Consequently, we are not able to test whether selection bias took place. This approach to recruit participants via social media channels also led to a relatively high number of participants who started the questionnaire and stopped or who did not complete the questionnaire with care. Though we are confident we removed these cases by studying suspicious and swift answering patterns, future research should find and recruit participants through alternative routes (e.g., inviting all employees of a large company or hospital to participate).

## Conclusion

**Fig. 3:**
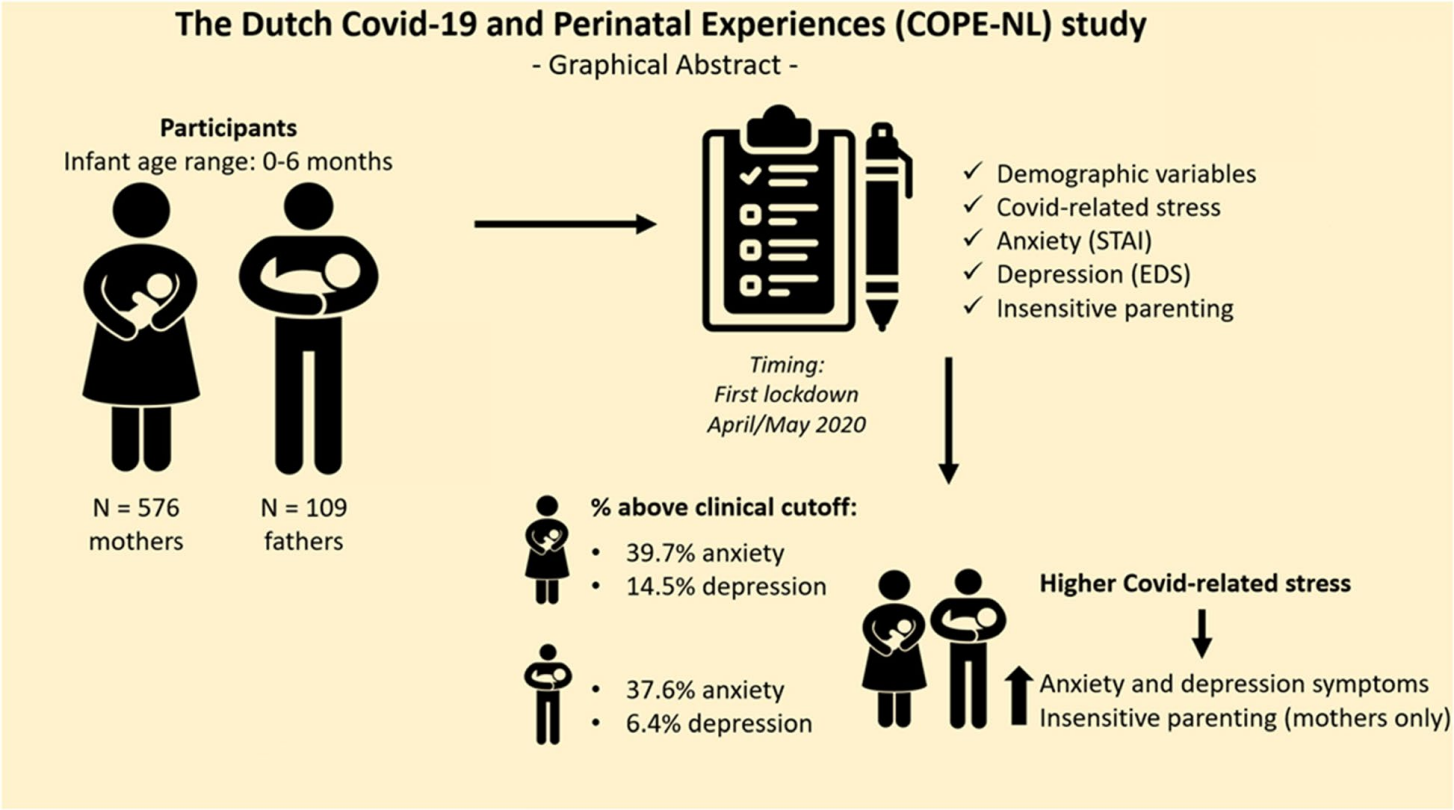
Graphical abstract. Overview of the study design and results

The results of this study emphasize the strain of the Covid-19 pandemic on young parents’ mental health in the Netherlands during its first lockdown. Though it was already clear that the Covid-19 pandemic has put an unprecedented pressure on families with children, it was unknown to what extent parents during the early postpartum period were affected. First of all (see also Fig. 3), we observed high percentages of clinically relevant mental health problems in mothers and fathers (i.e., mothers: 39.7% anxiety, 14.5% depression; fathers: 37.6% anxiety, 6.4% depression) and identified three types of Covid-19 related stress: general Covid-19 related worries, worries related to work and finances, and worries related to disruptions in social support. Mothers experienced more general Covid-19 worries, and worries related to disruptions in social support, compared to fathers. Moreover, mothers and fathers experienced the least worries related to disruptions in social support, which is a remarkable finding given that our results also indicate that especially these social support worries predicted worse mental health in parents and increased insensitive parenting in mothers. Covid-19 restrictions that put pressure on the social support network of young parents, such as social distancing, limited number of social contacts, and limited access to daycare, may impact parental mental health and, in turn, parenting practices in mothers. We could not replicate the latter finding in fathers, potentially due to a lower sample size in fathers. The results of this study can be used to inform policymakers and professionals about the burden and consequences of lockdown measures on young families. The results also indicate that special attention should be devoted to young parents’ lack of social support in times of crisis. Offering these parents emotional and instrumental support during the pandemic may be beneficial to improve their mental health and protect their parenting quality. Future studies should investigate factors that worsen or improve parental mental health (e.g., the role of (social) media coverage and crisis communication practices [63, 64];) and interventions stimulating parenting quality during times of crisis (e.g., an online mindful parenting training [65];). Future research should also replicate our findings in other countries, as countries managed the crisis and infections very differently with, for example, differences in lockdown strategies and attention paid to the consequences of the pandemic for citizens’ mental health (e.g., [66, 67]). As insensitive parenting early in life carries a risk for poorer child outcomes later, the mental health burden of the Covid-19 pandemic might not only have affected parents, but also the next generation; a question that also remains for future research.

## Data Availability

The dataset generated and analyzed during the current study is not publicly available because the participants did not gave permission to do so, but the dataset is available from the corresponding author on reasonable request.

## References

[1] Shuja KH, Shahidullah, Aqeel M, Khan EA, Abbas J (2020). Letter to highlight the effects of isolation on elderly during COVID-19 outbreak. Int J Geriatr Psychiatry.

[2] Aqeel M, Abbas J, Shuja KH, Rehna T, Ziapour A, Yousaf I, et al. The influence of illness perception, anxiety and depression disorders on students mental health during COVID-19 outbreak in Pakistan: a web-based cross-sectional survey. Int J Hum Rights Healthc. 2021. Publication ahead of print.

[3] Maqsood A, Abbas J, Rehman G, Mubeen R (2021). The paradigm shift for educational system continuance in the advent of COVID-19 pandemic: mental health challenges and reflections. Curr Res Behav Sci.

[4] Romero E, López-Romero L, Domínguez-Álvarez B, Villar P, Gómez-Fraguela JA (2020). Testing the effects of COVID-19 confinement in Spanish children: the role of Parents' distress, emotional problems and specific parenting. Int J Environ Res Public Health.

[5] Marchetti D, Fontanesi L, Mazza C, Di Giandomenico S, Roma P, Verrocchio MC (2020). Parenting-related exhaustion during the Italian COVID-19 lockdown. J Pediatr Psychol.

[6] Giannotti M, Mazzoni N, Bentenuto A, Venuti P, de Falco S. Family adjustment to COVID-19 lockdown in Italy: parental stress, coparenting, and child externalizing behavior. Fam Process. 2021(00):1–19. 10.1111/famp.12686 PMC8444949 34195986

[7] Hiraoka D, Tomoda A. Relationship between parenting stress and school closures due to the COVID-19 pandemic. Psychiatry Clin Neurosci. 2020. 10.1111/pcn.13088. 10.1111/pcn.13088 PMC7323183 32779846

[8] Chung G, Lanier P, Wong PYJ. Mediating effects of parental stress on harsh parenting and parent-child relationship during coronavirus (COVID-19) pandemic in Singapore. J Fam Violence. 2020:1–12. 10.1007/s10896-020-00200-1 PMC7467635 32895601

[9] Taubman-Ben-Ari O, Ben-Yaakov O, Chasson M (2021). Parenting stress among new parents before and during the COVID-19 pandemic. Child Abuse Negl.

[10] Racine N, Hetherington E, McArthur BA, McDonald S, Edwards S, Tough S, et al. Maternal depressive and anxiety symptoms before and during the COVID-19 pandemic in Canada: a longitudinal analysis. Lancet Psychiatry. 2021;8(5):405–415. 10.1016/S2215-0366(21)00074-2 PMC8824360 33773109

[11] Riecher-Rössler A, Hofecker Fallahpour M (2003). Postpartum depression: do we still need this diagnostic term?. Acta Psychiatr Scand.

[12] Skalkidou A, Poromaa IS, Iliadis SI, Huizink AC, Hellgren C, Freyhult E, Comasco E (2019). Stress-related genetic polymorphisms in association with peripartum depression symptoms and stress hormones: a longitudinal population-based study. Psychoneuroendocrinology.

[13] Dennis C-L, Ross L (2005). Relationships among infant sleep patterns, maternal fatigue, and development of depressive symptomatology. Birth.

[14] Batra K, Pharr J, Olawepo JO, Cruz P (2020). Understanding the multidimensional trajectory of psychosocial maternal risk factors causing preterm birth: a systematic review. Asian J Psychiatr.

[15] Ketner SL, Gravesteijn C, Verschuur MJ (2019). Transition to parenthood: it does not get easier the next time. Exploring ways to support well-being among parents with newborns. J Fam Soc Work.

[16] Wee KY, Skouteris H, Pier C, Richardson B, Milgrom J (2011). Correlates of ante- and postnatal depression in fathers: a systematic review. J Affect Disord.

[17] Leigh B, Milgrom J (2008). Risk factors for antenatal depression, postnatal depression and parenting stress. BMC Psychiatry.

[18] Benoit D (2004). Infant-parent attachment: definition, types, antecedents, measurement and outcome. Paediatr Child Health.

[19] Leppänen JM, Nelson CA (2009). Tuning the developing brain to social signals of emotions. Nat Rev Neurosci.

[20] Bates E, Dick F (2002). Language, gesture, and the developing brain. Dev Psychobiol.

[21] Koss KJ, Gunnar MR (2018). Annual research review: early adversity, the hypothalamic-pituitary-adrenocortical axis, and child psychopathology. J Child Psychol Psychiatry.

[22] Stein A, Pearson RM, Goodman SH, Rapa E, Rahman A, McCallum M, Howard LM, Pariante CM (2014). Effects of perinatal mental disorders on the fetus and child. Lancet.

[23] Ainsworth MDS, Blehar MC, Waters E, Wall S (1978). Patterns of attachment: a psychological study of the strange situation: Lawrence Erlbaum.

[24] Smith M (2004). Parental mental health: disruptions to parenting and outcomes for children. Child Fam Soc Work.

[25] Ahun MN, Côté SM (2019). Maternal depressive symptoms and early childhood cognitive development: a review of putative environmental mediators. Arch Womens Ment Health.

[26] Choi KR, Records K, Low LK, Alhusen JL, Kenner C, Bloch JR, Premji SS, Hannan J, Anderson CM, Yeo S (2020). Promotion of Maternal–Infant Mental Health and Trauma-Informed Care During the COVID-19 Pandemic. J Obstet Gynecol Neonatal Nurs.

[27] Prime H, Wade M, Browne DT (2020). Risk and resilience in family well-being during the COVID-19 pandemic. Am Psychol.

[28] VanTieghem M, Thomason ME, Graham A, Sullivan EL, Vatalaro T, Espinoza-Heredia C, Lenniger C, van den Heuvel MI. COVID-19 and perinatal experiences study. Retrieved from osfio/uqhcv 2020, November 23.

[29] Kaiser HF (1974). An index of factorial simplicity. Psychometrika.

[30] Kaiser HF (1970). A second generation little jiffy. Psychometrika.

[31] Bartlett MS (1954). A note on the multiplying factors for various χ<sup>2</sup> approximations. J R Stat Soc Ser B Methodol.

[32] Pop VJ, Komproe IH, van Son MJ (1992). Characteristics of the Edinburgh post Natal depression scale in the Netherlands. J Affect Disord.

[33] Cox JL, Holden JM, Sagovsky R (1987). Detection of postnatal depression. Development of the 10-item Edinburgh postnatal depression scale. Br J Psychiatry.

[34] Matthey S, Henshaw C, Elliott S, Barnett B (2006). Variability in use of cut-off scores and formats on the Edinburgh postnatal depression scale: implications for clinical and research practice. Arch Womens Ment Health.

[35] Matthey S, Barnett B, Ungerer J, Waters B (2000). Paternal and maternal depressed mood during the transition to parenthood. J Affect Disord.

[36] Matthey S, Barnett B, Kavanagh DJ, Howie P (2001). Validation of the Edinburgh postnatal depression scale for men, and comparison of item endorsement with their partners. J Affect Disord.

[37] Leung BM, Letourneau NL, Giesbrecht GF, Ntanda H, Hart M (2017). Predictors of postpartum depression in partnered mothers and fathers from a longitudinal cohort. Community Ment Health J.

[38] Van der Ploeg HM, Defares PB, Speilberger CD (2000). Handleiding bij de Zelf-beoordelings Vragenlijst ZBV: Een nederlandstalige bewerking van de Speilberger State-Trait Anxiety Inventory, STAI-DY.

[39] Spielberger CD (1983). State-trait anxiety inventory for adults (STAI-AD): APA PsycTests.

[40] Dennis CL, Coghlan M, Vigod S (2013). Can we identify mothers at-risk for postpartum anxiety in the immediate postpartum period using the state-trait anxiety inventory?. J Affect Disord.

[41] van Ijzendoorn MH, Bakermans-Kranenburg MJ, Coughlan B, Reijman S (2020). Annual research review: umbrella synthesis of meta-analyses on child maltreatment antecedents and interventions: differential susceptibility perspective on risk and resilience. J Child Psychol Psychiatry.

[42] Dubowitz H, Kim J, Black MM, Weisbart C, Semiatin J, Magder LS (2011). Identifying children at high risk for a child maltreatment report. Child Abuse Negl.

[43] Howard LM, Molyneaux E, Dennis C-L, Rochat T, Stein A, Milgrom J (2014). Non-psychotic mental disorders in the perinatal period. Lancet.

[44] Faul F, Erdfelder E, Lang AG, Buchner A (2007). G*power 3: a flexible statistical power analysis program for the social, behavioral, and biomedical sciences. Behav Res Methods.

[45] Koelewijn JM, Sluijs AM, Vrijkotte TGM (2017). Possible relationship between general and pregnancy-related anxiety during the first half of pregnancy and the birth process: a prospective cohort study. BMJ Open.

[46] Tharner A, Luijk MPCM, van Ijzendoorn MH, Bakermans-Kranenburg MJ, Jaddoe VWV, Hofman A, Verhulst FC, Tiemeier H (2012). Maternal lifetime history of depression and depressive symptoms in the prenatal and early postnatal period do not predict infant–mother attachment quality in a large, population-based Dutch cohort study. Attach Hum Dev.

[47] Missler M, van Straten A, Denissen J, Donker T, Beijers R (2020). Effectiveness of a psycho-educational intervention for expecting parents to prevent postpartum parenting stress, depression and anxiety: a randomized controlled trial. BMC Pregnancy Childbirth.

[48] Ceulemans M, Hompes T, Foulon V. Mental health status of pregnant and breastfeeding women during the COVID-19 pandemic: a call for action. Int J Gynecol Obstet. 2020; n/a(n/a). 10.1002/ijgo.13295 32620037

[49] Lebel C, MacKinnon A, Bagshawe M, Tomfohr-Madsen L, Giesbrecht G (2020). Elevated depression and anxiety symptoms among pregnant individuals during the COVID-19 pandemic. J Affect Disord.

[50] Vacaru S, Beijers R, Browne PD, Cloin M, van Bakel H, van den Heuvel MI, de Weerth C (2021). The risk and protective factors of heightened prenatal anxiety and depression during the COVID-19 lockdown. Sci Rep.

[51] Brown SM, Doom JR, Lechuga-Peña S, Watamura SE, Koppels T. Stress and parenting during the global COVID-19 pandemic. Child Abuse Negl. 2020;110:104699. 10.1016/j.chiabu.2020.104699 PMC7440155 32859394

[52] Oates M (2003). Suicide: the leading cause of maternal death. Br J Psychiatry.

[53] Goodman JH, Watson GR, Stubbs B (2016). Anxiety disorders in postpartum women: a systematic review and meta-analysis. J Affect Disord.

[54] Grant K-A, McMahon C, Austin M-P (2008). Maternal anxiety during the transition to parenthood: a prospective study. J Affect Disord.

[55] Fokkema CM, de Valk HAG, de Beer JAA, van Duin C (2008). The Netherlands: childbearing within the context of a "Poldermodel" society. Demogr Res.

[56] McGoron L, Riley MR, Scaramella LV (2020). Cumulative socio-contextual risk and child abuse potential in parents of young children: can social support buffer the impact?. Child Fam Soc Work.

[57] Hughes C, Devine RT, Foley S, Ribner AD, Mesman J, Blair C (2020). Couples becoming parents: trajectories for psychological distress and buffering effects of social support. J Affect Disord.

[58] Lee SJ, Ward KP, Chang OD, Downing KM. Parenting activities and the transition to home-based education during the COVID-19 pandemic. Child Youth Serv Rev. 2020;122:105585. 10.1016/j.childyouth.2020.105585 PMC7553006 33071407

[59] Morelli M, Cattelino E, Baiocco R, Trumello C, Babore A, Candelori C, Chirumbolo A (2020). Parents and children during the COVID-19 lockdown: the influence of parenting distress and parenting self-efficacy on Children’s emotional well-being. Front Psychol.

[60] Janssen LHC, Kullberg M-LJ, Verkuil B, van Zwieten N, Wever MCM, van Houtum LAEM, Wentholt WGM, Elzinga BM (2020). Does the COVID-19 pandemic impact parents’ and adolescents’ well-being? An EMA-study on daily affect and parenting. PLoS One.

[61] Liu CH, Mittal L, Erdei C. COVID-19-related health worries compound the psychiatric distress experienced by families of high-risk infants. J Perinatol. 2021;41:1191–1195. 10.1038/s41372-021-01000-1 PMC7928184 33658613

[62] Leach LS, Bennetts SK, Giallo R, Cooklin AR (2019). Recruiting fathers for parenting research using online advertising campaigns: evidence from an Australian study. Child Care Health Dev.

[63] Su Z, McDonnell D, Wen J, Kozak M, Abbas J, Šegalo S, Li X, Ahmad J, Cheshmehzangi A, Cai Y (2021). Mental health consequences of COVID-19 media coverage: the need for effective crisis communication practices. Glob Health.

[64] Abbas J, Aman J, Nurunnabi M, Bano S. The impact of social media on learning behavior for sustainable education: evidence of students from selected universities in Pakistan. Sustainability. 2019;11(6).

[65] Boekhorst MGBM, Hulsbosch LP, Nyklíček I, Spek V, Kastelein A, Bögels S, Pop VJM, Potharst ES (2020). An online mindful parenting training for mothers raising toddlers: assessment of acceptability, effectiveness, and personal goals. Mindfulness.

[66] Yoosefi Lebni J, Abbas J, Moradi F, Salahshoor MR, Chaboksavar F, Irandoost SF, Nezhaddadgar N, Ziapour A (2020). How the COVID-19 pandemic effected economic, social, political, and cultural factors: a lesson from Iran. Int J Soc Psychiatry.

[67] Wang C, Wang D, Abbas J, Duan K, Mubeen R. Global financial crisis, smart lockdown strategies, and the COVID-19 spillover impacts: a global perspective implications from Southeast Asia. Front Psychiatry. 2021;12:643783. 10.3389/fpsyt.2021.643783 PMC8446391 34539457

